# Non-alcoholic fatty liver disease: leading the fight in primary care

**DOI:** 10.3399/bjgp22X720917

**Published:** 2022-10-30

**Authors:** Mark D Theodoreson, Richard Darnton, Ian Rowe, Richard Parker

**Affiliations:** Leeds Liver Unit, Leeds Teaching Hospitals NHS Trust, Leeds.; Department of Public Health and Primary Care, University of Cambridge, Cambridge.; Leeds Liver Unit, Leeds Teaching Hospitals NHS Trust, Leeds; Leeds Institute for Medical Research, University of Leeds, Leeds.; Leeds Liver Unit, Leeds Teaching Hospitals NHS Trust, Leeds.

## NON-ALCOHOLIC FATTY LIVER DISEASE IN PRIMARY CARE

GPs are no strangers to non-alcoholic fatty liver disease (NAFLD) and are commonly faced with non-specific liver function tests (LFTs) or an incidental finding of steatosis on ultrasound scan (USS). Interpreting these results and conveying their significance to the patient can be a challenge, with huge variation in practice.

NAFLD is a spectrum of liver abnormalities from simple fat deposition (steatosis) to inflammation (non-alcoholic steatohepatitis, NASH). Steatohepatitis causes persistent hepatocellular inflammation leading to fibrosis that can, in some individuals, progress to cirrhosis. The disease burden of NAFLD is rising, now the most common liver disease globally, affecting up to 46% of all adults.^1^ Importantly, only 0.5% of patients are expected to progress to cirrhosis, which carries a risk of liver failure.^2^ Patients with NAFLD are at greater risk of all-cause mortality and, for most patients, the risk of non-hepatic ill health greatly outweighs the risk of liver-related morbidity.^1^^–^^3^ NAFLD is overwhelmingly associated with modifiable lifestyle factors, with obesity and metabolic syndrome the key drivers.^1^ This review describes how and when to make a diagnosis of NAFLD, the use of fibrosis markers to identify patients at risk of significant disease, and appropriate follow-up.

## CURRENT PRACTICE IN PRIMARY CARE

The majority of diagnoses occur in primary care,^4^ where clinicians can identify those at risk, initiate investigations, and advise regarding modifiable lifestyle factors. It is therefore essential that GPs are knowledgeable about the investigations available locally and how to action the results. However, there is little concordance of current guidelines, and this reduces certainty in decision making.

## PRESENTATION

NAFLD usually presents with either deranged LFTs or an incidental finding on an USS; however, the majority of patients with NAFLD have normal LFTs. The European Association for the Study of the Liver (EASL)^1^ advises screening for NAFLD in those with metabolic risk factors; however, there is debate about the usefulness of this approach given that the majority of patients do not progress to clinically significant disease.^3^ National Institute for Health and Care Excellence (NICE) guidelines do not support screening.^6^ The British Society of Gastroenterology (BSG) suggests that further studies are needed to establish cost-effectiveness of case finding in NAFLD.^5^ When considering the aetiology of deranged LFTs, clinicians need to remain aware that there are increasing cases of NAFLD in children and young adults, and in up to 7% of lean individuals.^7^

## ABNORMAL TESTS: WHAT NEXT?

Current recommendations are for patients with deranged LFTs, regardless of metabolic risk factors, to have a full non-invasive liver screen.^5^ This screen can help rule out other pathologies but provides limited information regarding prognosis. Understanding the stage of liver disease, and the risk of poor outcomes, is done using liver fibrosis testing. If LFTs are <4 × upper limit of normal (ULN), with no concerning features, it would be reasonable to repeat LFTs at 3–6 months following lifestyle modification advice.

## LIVER AETIOLOGY SCREEN

The BSG, with representation from the Royal College of General Practitioners,^5^ have developed an algorithm for clinicians to use when determining the aetiology of liver pathology. A standard liver screen should include an USS, viral screen, autoimmune screen, and ferritin levels.^5^ The use of liver USS in assessing NAFLD is questionable. Sensitivity is limited when hepatic fat is less than 30%, and it adds no prognostic information.^5^ USS can help rule out other liver or biliary disease. In a patient with risk factors for NAFLD +/− steatosis, deranged LFTs, and an otherwise normal liver screen then a diagnosis of NAFLD can reliably be made. If there is a history of high alcohol intake, then alcohol-related fatty liver must be considered.

Serum fibrosis markers should be used to rule out significant fibrosis.^1^ Despite the high prevalence of NAFLD, the majority will never progress to advanced fibrosis. Simple fibrosis scores are an acceptable way to stratify risk and filter those needing referral to secondary care; however, cost-effectiveness needs establishing.^5^ Though non-invasive tests lack the sensitivity and specificity to differentiate non-alcoholic steatohepatitis from simple steatosis,^8^ they can reliably prognosticate future liver-related events and mortality.^4^

FIB-4 and NAFLD fibrosis score (NFS) are recommended by the BSG for first-line screening. They are widely available, using routine information, ensuring they are quick and inexpensive to perform. A FIB-4 score of <1.3 (<2.0 if >65 years) or NFS <−1.455 (<−0.120 if >65 years) indicates low risk of advanced fibrosis and can be managed in primary care. A FIB-4 score >3.25 or NFS >0.675 should trigger a referral for specialist opinion. If the value falls in the ‘grey zone’ between these, an enhanced liver fibrosis (ELF) test or Fibroscan should be performed for further evaluation. NICE guidelines^6^ advise using the ELF test when screening for advanced fibrosis; however, other validated fibrosis scores are available, as well as imaging modalities such as transient elastography (TE), for example, Fibroscan.

A high ELF score is associated with increased risk of liver-related events.^8^ ELF has been shown to have a sensitivity and specificity of 74% and 73% respectively.^9^ If ELF is not available, then TE should be considered. EASL advises that a combination of biomarkers and TE may improve diagnostic accuracy, with the combination of FIB-4 and TE achieving similar prognostic accuracy as a biopsy.^1^^,^^9^

## NON-ALCOHOLIC FATTY LIVER DISEASE MANAGEMENT: A PRIMARY CARE PERSPECTIVE

**Figure 1.**
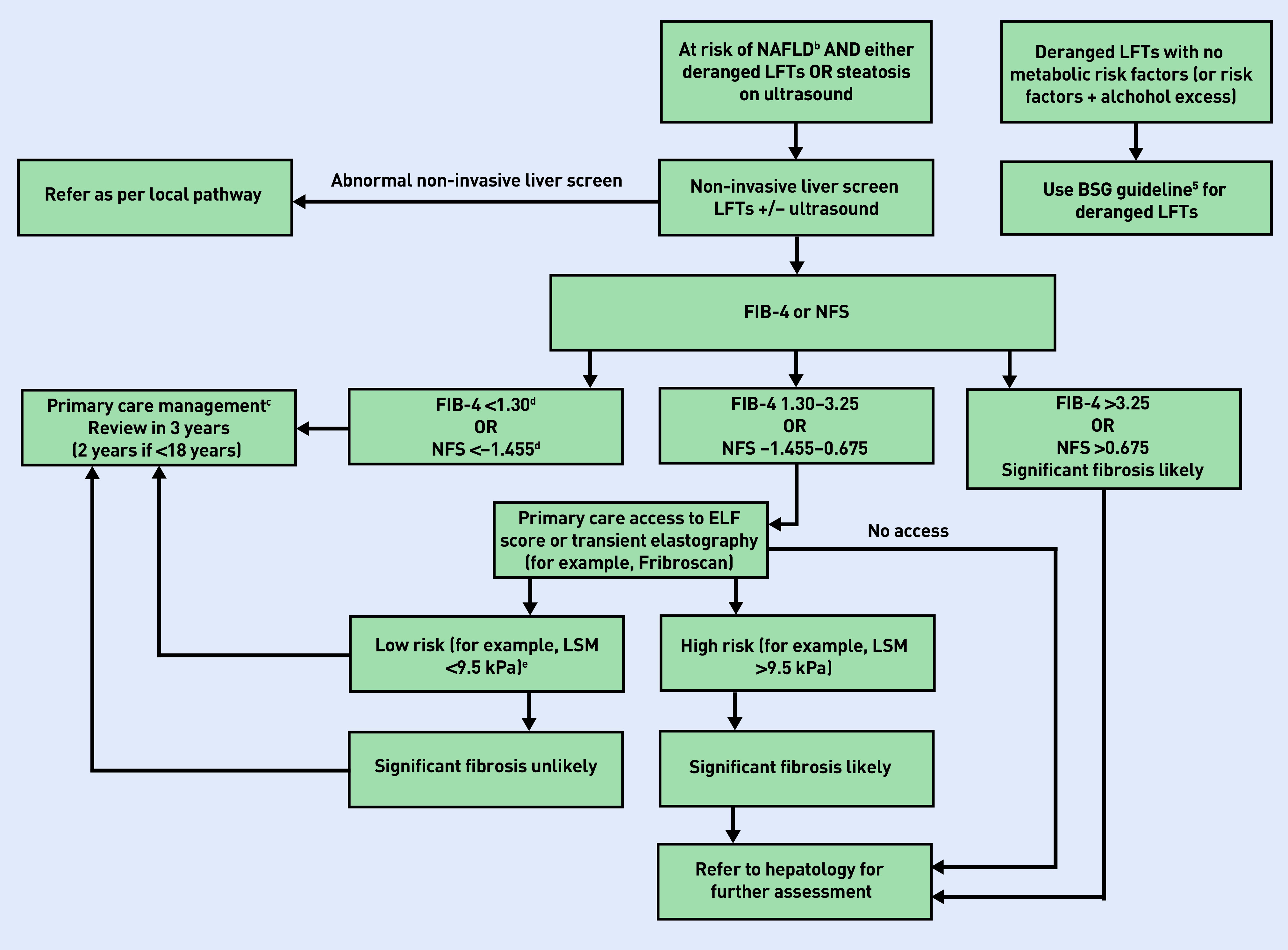
*Diagnosis algorithm.^a^* *^a^There is no common consensus between guidelines. This algorithm is the authors’ design and an amalgamation of current literature and guidelines.^1^^,^^5^^,^^6^ Clinicians should refer to their local pathways where these exist. ^b^Metabolic syndrome if >3 of 5 present: impaired fasting glucose or T2DM, hypertriglyceridaemia, low HDL cholesterolaemia, increased waist circumference/raised BMI >30, or hypertension. ^c^Assess cardiovascular risk, diet and exercise advice, alcohol reduction, diabetes management. ^d^Note if aged >65 lower cut-offs are: NFS = <–0.12; FIB-4 = <2.0. ^e^Precise thresholds vary between commissioned pathways. BMI = body mass index. BSG = British Society of Gastroenterology. ELF = enhanced liver fibrosis. HDL = high-density lipoprotein. LFT = liver function test. LSM = liver stiffness measurement. NAFLD = non-alcoholic fatty liver disease. NFS = NAFLD fibrosis score. T2DM = type 2 diabetes mellitus.*

There is currently no approved treatment for NAFLD and the number of patients experiencing adverse liver outcomes is small, raising questions on the benefits of population screening until there are effective, evidence-based therapies available to prevent progression.^3^ The focus should be on a proactive approach to managing metabolic risk factors to reduce all-cause morbidity and mortality,^3^ as opposed to case finding and diagnosis. Box 1 lists some important questions for GPs. No specific diet is recommended; rather, patients should be encouraged to achieve their weight-loss goal in a sustainable way. If indicated by cardiovascular risk, statin therapy should continue,^6^ only stopping if LFTs double within 3 months of initiation. If a patient has NAFLD but does not meet the threshold for referral, reassessment should occur every 3 years. A diagnostic and referral pathway based on the latest evidence has been developed by the authors of this article (Figure 1); however, clinicians should be familiar with local guidelines as referral pathways vary across integrated care systems.

**Box 1.** Common questions

Q: I have an USS report with an incidental finding of fatty liver — do I code the patient as having NAFLD? A: Not yet — just as not all coronary plaque is considered IHD, not all fatty liver on USS is NAFLD. If there are associated deranged LFTs or raised fibrosis markers then NAFLD can be diagnosed (in the absence of other pathology). NB: though not strictly NAFLD, individual CCGs may wish this coded as such for funding reasons. Q: My patient has a fatty liver and a mildly raised ALT (<4 × ULN). Their FIB-4 score is 0.9. Do I need to refer? A: This patient likely has NAFLD but a low risk of progression. You should discuss lifestyle modifications and aim to optimise comorbid metabolic risk factors. Re-assess FIB-4 in 2–3 years. Q: My patient has mildly deranged LFTs (<4 × ULN) and evidence of fibrosis on Fibroscan. Can I continue their statin? A: Yes. Statins are safe and will reduce cardiovascular risk.

*ALT = alanine aminotransferase. CCG = clinical commissioning group. IHD = ischaemic heart disease. LFTs = liver function tests. NAFLD = non-alcoholic fatty liver disease. ULN = upper limit of normal. USS = ultrasound scan.*

## SUMMARY

NAFLD is primarily a metabolic disease, heavily influenced by lifestyle factors. GPs’ exposure to patients at risk is on an upward trajectory. Physicians must consider the burden of testing for these patients and the proactive management of risk factors to reduce future events.
